# Supplementary figures and images for: Type I interferon-dependent CCL4 is induced by a cGAS/STING pathway that bypasses viral inhibition and protects infected tissue, independent of viral burden
Source: PLoS Pathog. 2019 Oct 11;15(10):e1007778. doi: 10.1371/journal.ppat.1007778 (PMC6808495; doi:10.1371/journal.ppat.1007778)

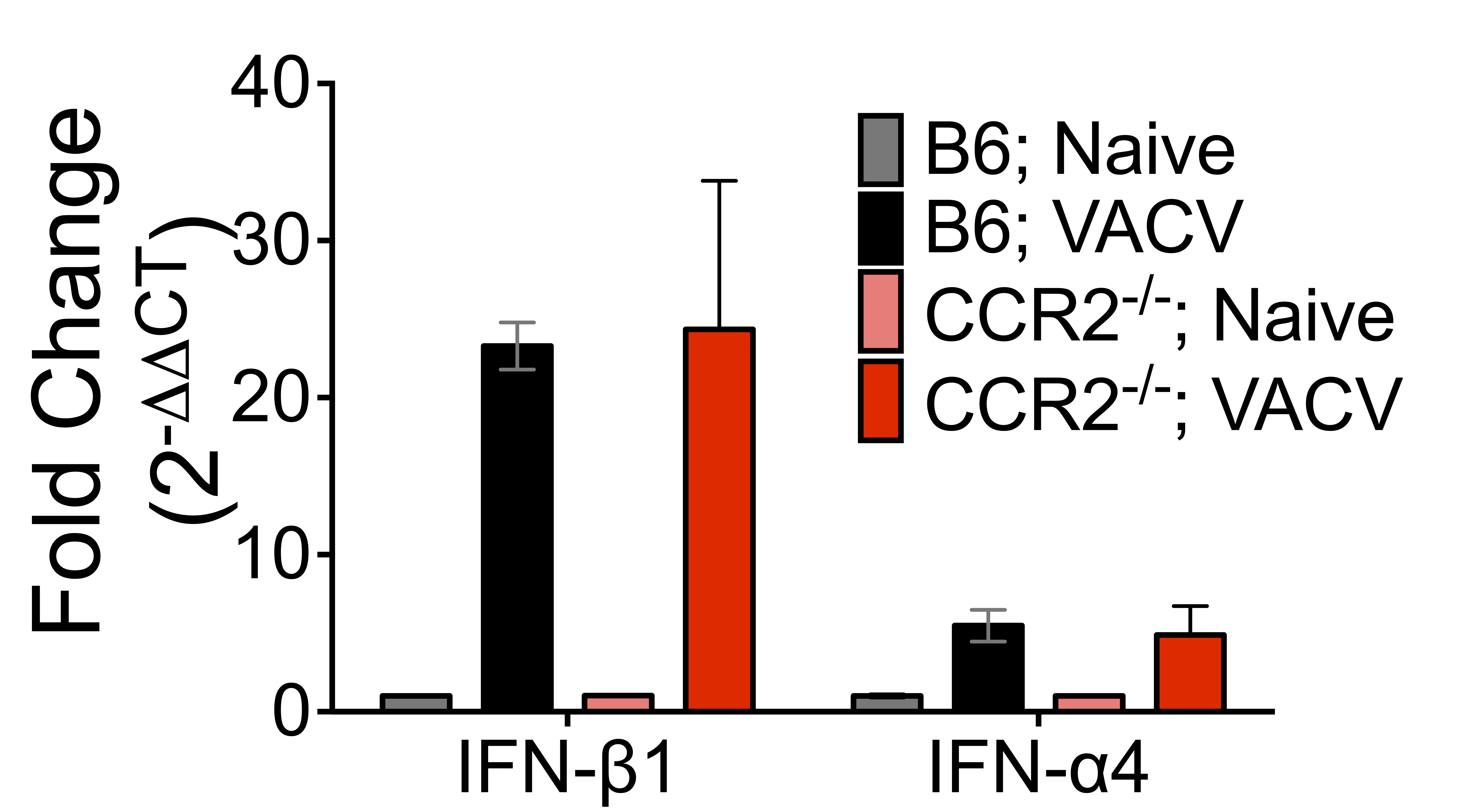

Supplement: S1 Fig — (A) Expression of T1-IFN subtypes IFN-β1 and α4 in ear tissue of wild-type (B6) vs CCR2-/- mice, in which recruitment of inflammatory monocytes to the VACV infected ear is completely ablated (55). mRNA was purified and expression levels from uninfected mice, or at 5 dpi, measured by RT-qPCR. (TIFF) [file ppat.1007778.s001.tiff]
